# Human exposome assessment platform

**DOI:** 10.1097/EE9.0000000000000182

**Published:** 2021-12-03

**Authors:** Roxana Merino Martinez, Heimo Müller, Stefan Negru, Alex Ormenisan, Laila Sara Arroyo Mühr, Xinyue Zhang, Frederik Trier Møller, Mark S. Clements, Zisis Kozlakidis, Ville N. Pimenoff, Bartlomiej Wilkowski, Martin Boeckhout, Hanna Öhman, Steven Chong, Andreas Holzinger, Matti Lehtinen, Evert-Ben van Veen, Piotr Bała, Martin Widschwendter, Jim Dowling, Juha Törnroos, Michael P. Snyder, Joakim Dillner

**Affiliations:** a Karolinska Institutet, Stockholm, Sweden; b Medical University Graz, Graz, Austria; c CSC—IT Center for Science Ltd, Espoo, Finland; d Logical Clocks AB, Stockholm, Sweden; e Stanford University, Stanford, CA; f Infectious Disease Epidemiology and Prevention, Statens Serum Institut, Copenhagen, Denmark; g International Agency for Research on Cancer, World Health Organization, Lyon, France; h Faculty of Medicine, University of Oulu, Oulu, Finland; i Tampere University, Tampere, Finland; j Danish National Biobank, Statens Serum Institut, Copenhagen, Denmark; k MLCF (Stichting MLC Foundation), The Hague, The Netherlands; l Biobank Borealis of Northern Finland, Oulu University Hospital, Oulu, Finland; m University of Warsaw, Warsaw, Poland; n Research Institute for Biomedical Aging Research, Universität Innsbruck, Innsbruck, Austria.

**Keywords:** Exposome, Cohort, PaaS, IaaS, AI, Bioinformatics, Machine learning, Data management, FAIR

## Abstract

The Human Exposome Assessment Platform (HEAP) is a research resource for the integrated and efficient management and analysis of human exposome data. The project will provide the complete workflow for obtaining exposome actionable knowledge from population-based cohorts. HEAP is a state-of-the-science service composed of computational resources from partner institutions, accessed through a software framework that provides the world’s fastest Hadoop platform for data warehousing and applied artificial intelligence (AI). The software, will provide a decision support system for researchers and policymakers. All the data managed and processed by HEAP, together with the analysis pipelines, will be available for future research. In addition, the platform enables adding new data and analysis pipelines. HEAP’s final product can be deployed in multiple instances to create a network of shareable and reusable knowledge on the impact of exposures on public health.

**What This Project Adds**

The Human Exposome Assessment Platform will enable the standardized management and analysis of heterogeneous environmental exposure data. It provides a complete research resource for knowledge discovering through bioinformatics analyses, advanced statistics, and machine learning. The platform will be populated with data from large-scale population-based cohort studies on environmental exposures nested in organized cancer screening, a population based healthy childbearing cohort, vaccination trials and a nationwide consumer cohort. A pilot study will be carried out on maternity exposure by monitoring pregnant women using an innovative wearable exposome sensor to generate personal health profiling of the participants. Successful data management of diverse exposure data on the same informatics platform will provide a proof-of-principle on how collaborative, multinational exposome research can be conducted in a synergistic manner.

## Introduction

Exposome research can result in the design of cost-effective health interventions targeting environmental risk factors that affect human health. For instance, the age-adjusted incidence of chronic diseases such as cancer, is rising. The exposome risk factors behind this increment are not fully determined. An integrated research framework that efficiently provides streamlined tools for exploiting major technologies and disciplines involved in the research on exposome risk factor assessment, could dramatically contribute to identifying the environmental factors affecting human health.

The HEAP is developing a global research resource that integrates an Infrastructure as a Service (IaaS: computational resources services for storage and computation) and a Platform as a Service (PaaS: software services for managing and analyzing the data) for the efficient management and processing of massive data from geographically distributed large-scale population cohorts.

HEAP will enable the creation of collaborative networks towards the joint production of consistent and actionable knowledge to tackle the effects of exposome on health and society. As proof of concept, HEAP will provide data and knowledge about multiexposome assessment from large-scale population-based cohorts: (1) a nation-wide Human Papilloma Virus (HPV) vaccination cohort that provides knowledge about impact of HPV vaccination in the population; (2) in a subset of the HPV vaccination cohort, the impact of exposures on the health of pregnant women will be supported by real-time measures from wearable exposure sensors and metabolomics analyses; (3) a large cervical cancer screening cohort enabling studies on women’s health supported with systematic epigenomics and metagenomics analyses; (4) a nation-wide maternity cohort providing longitudinal data to enrich knowledge about women’s health; and (5) systematic collection of consumer purchase data from digital receipts, linked to health outcomes, which will enable the assessment of purchase-related exposures on the health in households.

**Figure 1.**
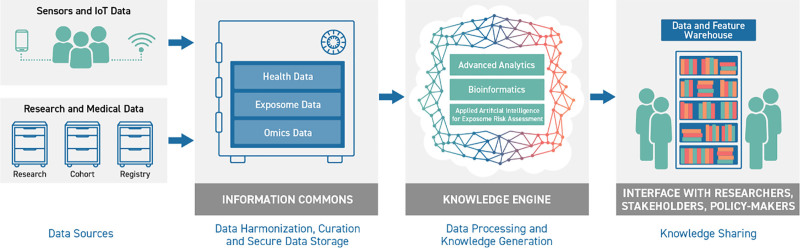
Conceptual model reflecting the data cycle in HEAP. From different data sources, the data is preprocessed and stored for analysis in the IC, the data are analyzed by the Knowledge Engine (KE) and made available to the stakeholders for predictions and interpretations.

The conceptual model of HEAP is illustrated in Figure 1.

## Project description

### Aim

The main objective of HEAP is to enable global collaborative exposome research towards cost-effective health interventions. This will be achieved through a research and technical platform that implements a robust ethic-legal framework and integrates advanced Information Communication Technology (ICT), state-of-the-science exposome measurement technologies and uniquely large longitudinal population-based cohorts that will provide valuable actionable knowledge as proof of concept. HEAP aims also to demonstrate the creation of personal exposome health profiling integrating into the platform an innovative wearable exposure sensor that collects airborne and toxic substances as well as particulate matters derived from virus, bacteria, fungal spores, animal debris, and plant pollens.

The final product from HEAP is an informatics platform that integrates innovative data management, state-of-the-art data analysis pipelines, Internet of Things (IoT) technology, advanced statistics and applied AI; and can be deployed in computer clusters and computing centers worldwide.

### HEAP frameworks

**Figure 2.**
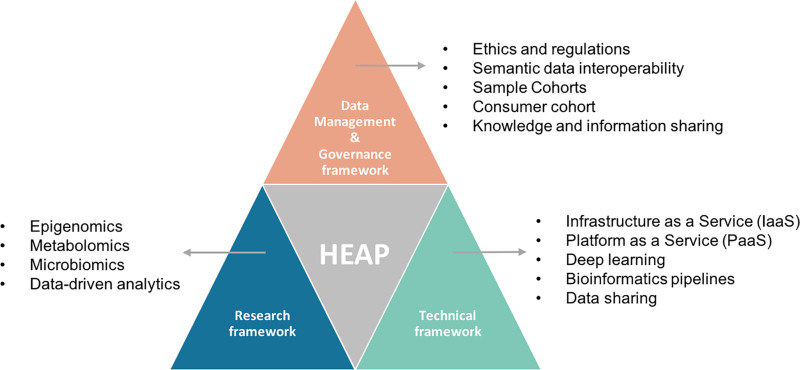
Three major frameworks are integrated into HEAP: Data management and Governance, Research and Technical, which provides the Information Communication Technology (ICT) resources, methods, and solutions to implement the data management and research in the HEAP platform.

HEAP is driven by three major frameworks as illustrated in Figure 2.

### HEAP management & governance framework

The management & governance framework covers all the issues related to semantic interoperability of the data following ethics and regulations, as well as dissemination and sharing of the produced knowledge.

The HEAP cohorts provide the relevant elements to design and implement a robust ethical and regulatory governance, and a standardized exposome data management system.

### HEAP cohorts and clinical studies

HEAP will carry out multiexposome analyses on three sustainable large-scale intervention cohorts: (1) The Swedish Cervical Cancer Screening Cohort with data and samples from about 1 million cervical screenings, about 0.5 million individual women—enrolling about 150,000 women per year, (2) The Finnish Community Randomized trial of HPV vaccination, which is the largest HPV vaccination trial in the world and has a very large associated sample cohort of oral gargles and cervical cells, (3) The Finnish Maternity Cohort containing data and samples from all pregnancies in Finland since 1983 with about 2 million pregnancies; and about 1 million unique women.^1–5^ The cohorts provide comprehensive data from samples and health data registries (including lifestyle, health and disease history, health costs and deaths) for integrated analysis of both internal and external exposure. Some of the pregnant women in the HPV vaccination Cohort (~100 pregnant women) will be followed using an innovative wearable exposure sensors system for the monitoring of multiple exposures and health measurements, to investigate how the exposome influences the health of the mother and the baby.

Finally, HEAP will pilot the entire process from recruitment to knowledge generation, systematic collection of consumer receipts and linkages to registries, to allow comprehensive analysis of the consumed exposomes impact on health or disease. The establishment of the large consumer cohort will become a resource for future research (the Danish Consumer Cohort).

### Consumer purchase data

Consumer purchase data (CPD) are emerging as a promising source to map exposures over prolonged timespans. One digital receipt corresponds to a yes/no questionnaire with well over 10 000 products available in each supermarket or store, replacing data from dietary questionnaires. This could provide the scale, coverage, objectivity, and long periods of dynamic data collection needed to reflect an individual’s exposome over time.^6–10^ This could allow researchers to model the consumed products impact on health and has already provide successful use in several food borne outbreak investigations.^11^ A Danish study on consumer patterns found that unemployment is associated with increased sugar content in the purchases, and intervention studies indicate that consumer data could provide meaningful feedback with potential to change consumer behavior.^12,13^

### HEAP research framework

HEAP research framework is dedicated to investigate, explore, and test state-of-the-science solutions for exposome data analysis focused on epigenomics, microbiomics, metabolomics, wearable sensors, advanced statistics, and Artificial Intelligence (AI). The high-quality solutions selected in this framework, are implemented in the HEAP platform to be available for the research community. The most relevant technologies applied to the proof of concept cohorts are explained below.

### Epigenomics

The epigenome can be thought of as a mechanism of cellular memory that records environmental exposures which accumulate during lifetime and which potentially explain predisposition to late-onset disease such as cancer and other chronic diseases. DNA methylation markers also serve as a good proxy for the replicative age of cells and tissues. The epigenome will be systematically measured using cervical cell samples continuously collected into the Swedish Cervical Screening cohort as well as collected within interventional clinical trials, which assess the effects of smoking cessation and intermittent fasting and caloric restriction.

### Microbiomics

Different bioinformatic pipelines targeting the study and analysis of the microbiome will be available as user-friendly solutions for researchers. Bioinformatics tools will be available through Hopsworks (https://www.logicalclocks.com) in a Hadoop/Spark environment. We are developing a bioinformatics solution based on our previous work, which will enable researchers to mine for different microorganisms present in a specimen with an increased speedup of at least 11× when using 23 nodes, compared with any sequential analysis pipeline executed on a single node.^14^ The solution covers the whole process for metagenomic analysis, from FASTQ raw files uploading, quality trimming, host (e.g., human) filtering, taxonomy classification of nonhost reads, de novo-assembly, and detection of highly divergent or yet unknown viruses. Furthermore, tools for transcriptomics (RNA sequencing) will also be available to understand the biologic significance (replication) for the different microorganisms and specific pipelines designed for human papillomavirus detection.

The microbiome will be comprehensively measured using subsets of the screening cohort and the resulting data analyzed using the HEAP analysis platform.

### Metabolomics and wearable exposome sensors

Traditional monitoring data on broad areas cannot reflect the complexity and dynamics of individual environmental exposures. Prof. Michael Snyder, from Stanford University, created a platform based on a wearable personal exposome monitor (PEM) device that captures and analyses a large amount of abiotic and biotic environmental contaminants.^15,16^ The monitoring devices previously published were costly (~USD 2,700) and contained battery-drain functionalities not suitable for daily monitoring. As part of HEAP, the PEM has been improved with a cartridge with hydrophilic (polyethersulfone) filter to capture the biologic components of particulate matter (PM) and a cartridge for sorbents which collect aerosol chemicals (both hydrophilic and hydrophobic). The improved PEM has twice the flow rate as before, which allows a larger number of particles to be collected. Other features of the device include measuring temperature, humidity, and GPS coordinates. We performed a proof-of-concept study to evaluate the performance of the device. The new device collects more biologic and chemical particles compared with the old device. The new PEM will be used on consented study participants to monitor their personal exposure over the course of the study (Table 1).

**Table 1.**
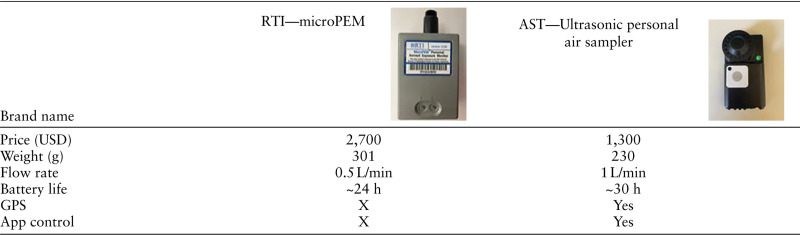
Technical specification comparison

**Figure 3.**
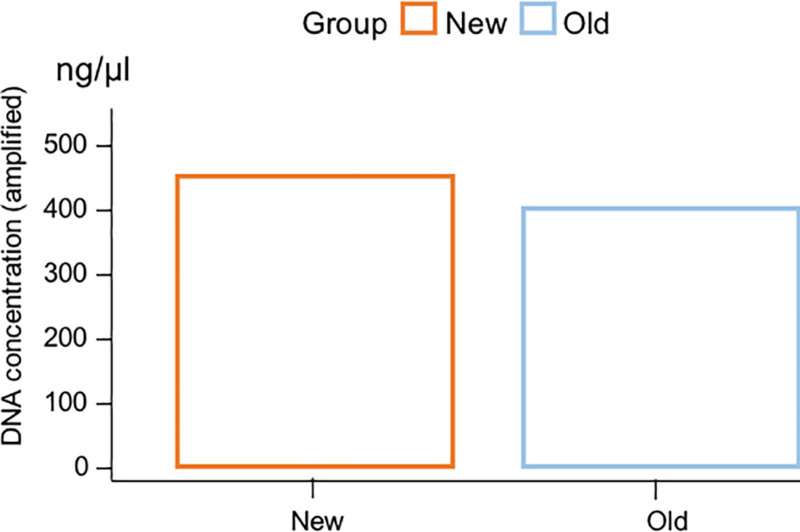
Comparison of DNA concentration collected by new and old PEM.

**Figure 4.**
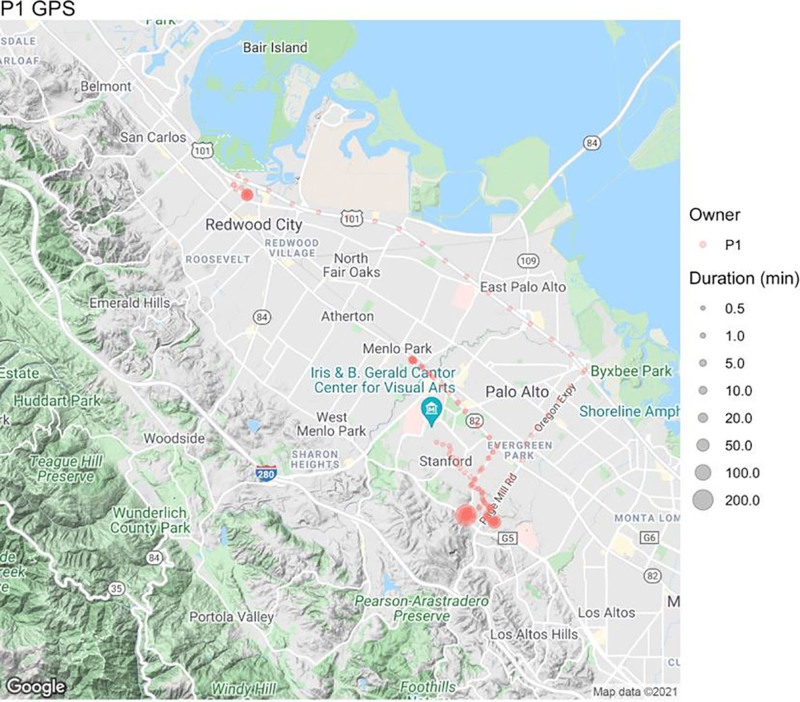
Example of GPS coordinates recorded by the new PEM.

The new PEM is suitable for daily exposure monitoring. Compared with the older device, the improved PEM has twice the flow rate (with the potential to reach four times the flow rate), hence collecting more materials (Figure 3), and a longer battery life. In addition, the improved PEM costs only half the price of the older one and can record GPS coordinates (Figure 4 shows an example).

### Advanced statistics analysis on HEAP

We have begun to develop tools for random effect modeling using automatic differentiation and variational approximations. Code has been developed for partially separable quasi-Newton methods,^17^ which is useful for optimization of models based on variational approximations; fast variational approximations for mixed models based on the probit link,^18^ and variational approximations for mixed generalized survival models.^19^ The next step in this process is to develop tools for joint modeling of longitudinal and survival outcomes.

Synthetic datasets from observed infections may offer a solution for complex pathogen occurrence pattern and interactions estimation. We have also explored viral occurrence patterns by developing log-linear models combined with Bayesian framework for network analysis. Based on modeling the probabilistic associations between observed data points, that is, HPV infections, we simulated HPV networks from the observed HPVs cohort datasets. Our analysis outperformed in precision all oversampling methods tested for simulating large synthetic viral prevalence dataset from observed data.^20^

We have also developed tools for multistate models based on ordinary differential equations coupled with their sensitivity equations for variance calculations. An application of these models was presented in 2020 and a methods article has been submitted.^21^ The source code is available on the Comprehensive R Archive Network.^22^ For applications based on microsimulation, we recently modeled for the cost-effectiveness of cervical cancer screening.^23^ These results have been presented to the National Board of Health and Welfare, and we plan to use this model to evaluate the upcoming Swedish cervical screening guidelines.

We have also contributed to a “disease wide association study,” where different causes of hospitalization and death were assessed by blood type.^24^ This study is relevant to HEAP, as it demonstrates how methods based on false discovery rates from genetic epidemiology can be applied to other epidemiologic study designs.

Finally, we contributed to an analysis of AI-based prostate histopathology.^25^ We showed that AI-based histology can perform as well as pathologists in determining which biopsy cores are expected to have cancer and in determining the grade of prostate cancer. We also wrote code to compare the performance of the different pathologists using average kappa statistics.^26^

### HEAP technical framework

The technical framework implements the management and the research frameworks into the HEAP informatics platform making possible that researchers, policymakers, and other stakeholders can manage and extract knowledge from exposome data.

The technical framework consists of (1) HEAP IaaS: distributed high-performance computational resources, (2) HEAP PaaS: a software platform that integrates the computing resources and enables secure management of exposome data, and (3) analysis pipelines implemented in the platform for the processing of harmonized and interoperable heterogeneous data. The software platform will apply AI and advanced statistics analyses for the continuous identification and evaluation of the roles of multiple exposures and its impact on human health.

**Figure 5.**
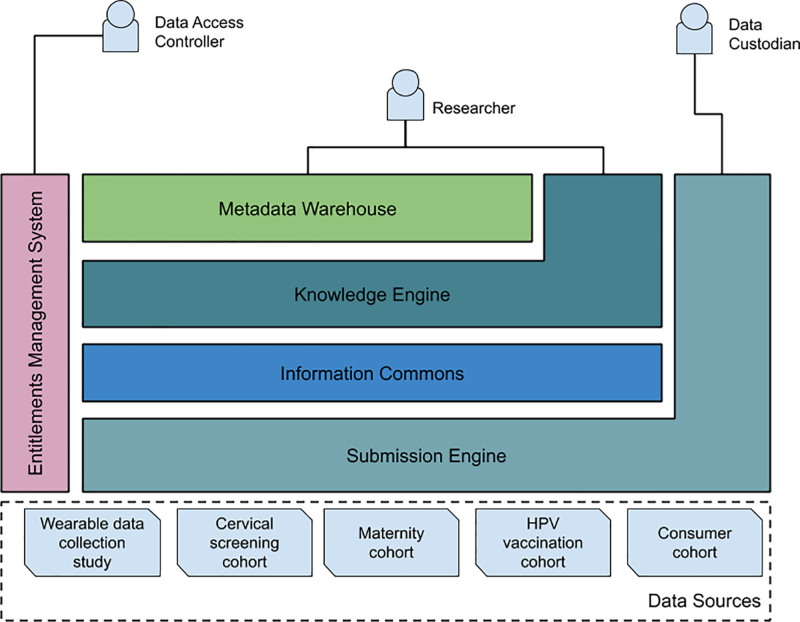
HEAP reference architecture components (RAC).

Figure 5 illustrates the HEAP Reference Architecture and its components as single instances.

### Submission engine

Interface with the information commons (IC) and enables the storing of sensitive data in an encrypted format.

### Information commons

Infrastructure to store data and information for current and future research.

### Knowledge engine

Software tools for data-driven analysis, bioinformatics, and machine learning including a feature store for training learning models.

### Metadata warehouse

Makes available metadata about the data stored in IC and metadata in the knowledge engine (KE).

### Entitlements management system

Manage access rights to resources in the IC and provides traceability of the data access rights granted. Manages authentication and authorization and validates secure standards for access delegation.

Considering the need for a distributed system, the IC, KE, metadata warehouse, and entitlements management system components can be scaled to multiple instances. The submission engine layer will be able to submit data to any of the instances of the IC.

### HEAP FAIR data management principles

HEAP follows the FAIR (Findable, Accessible, Interoperable, Reusable) principles to securely manage data from patients, pathogenic organisms, and from global genetic resources.^27^ HEAP will connect its IC to the European Open Science Cloud (EOSC) via consistent, FAIR annotation practices (including provenance information) and ensure that data publication meets ethical and legal requirements. Access to data and cloud services will be underpinned by a shared federated Life Science Authentication and Authorization Infrastructure (AAI). A common provenance information model enables meaningful reuse of data, as it allows to trace history of the biobank samples and data from the biologic material acquisition, through data generation, to data processing, and to meet requirements from General Data Protection Regulation (GDPR) and the Nagoya protocol on access and benefit sharing.^28^ These principles foster traceability, explainability, and causability and can support knowledge discovery, namely not only recognizing but also tracing.^29^ They can also provide a causal explanation of existing relationships and discovering new relationships in the underlying data. HEAP will train future research users to provide them with the skills needed for long-term operation and expansion of the data resources. The educational activities are based on MIABIS training on data collection for biologic samples of research institutes within the EU and LMICs.^30^

### Hopsworks

Hopsworks is the software platform that enables managing and analyzing data. It is the core of the HEAP platform, which interfaces the users with the software and the computational resources. Hopsworks stores and accesses files from a distributed file system, and runs jobs over multiple workers (script running is the background) running on different nodes through a resource manager. As machine learning (ML) support is paramount, Hopsworks provides managed access to multiple graphics processing units (GPUs) located over different nodes to the running applications. Additionally, the platform provides support to common data analytics tooling, ML libraries, stream processing, and metadata search and exploration. Hopsworks is an ecosystem of open-source libraries and frameworks, which is continuously upgraded to their most recent versions to provide optimum functionality to the users.

The Hopsworks platform is deployed as a testbed on the IaaS public cloud service (cPouta)^31^ at the IT Center for Science Ltd (CSC). Integration with the CSC sensitive data services^32^ (IC) has started with the integration of the remote authentication service. Hopsworks now provides a possible log-in mechanism and its own native user/password mechanism, as well as a CSC test user authentication service and Elixir AAI.^33^ This integrated authentication mechanism can already be used on the HEAP testbed.

Ongoing work includes integration with the CSC Sensitive Data Services and Resource Entitlement Management System (REMS).^34,35^ This integration will allow Hopsworks users and applications running on behalf of the user to access sensitive data stored in IC (provided via the CSC Sensitive Data Services) if the user is supposed to have access to it, as registered in REMS. The aim is also to make use of CSC IaaS private cloud service (ePouta),^36^ which is designed for processing sensitive data.

Hopsworks provides its own internal catalogue for metadata, but this was in a simple key-value format, where the value can be of string type. As part of the HEAP project, we have extended this metadata format to what we call “schematized tags.” Through the schematized tags mechanism, we have extended what types the value can take and this includes primitives, JSON objects, and arrays. Not only can the metadata value take these complex values, but the metadata is searchable through each of its component values. The schematized tags follow the JavaScript Object Notation (JSON) schemas.^37^ Besides the complex type that values can take, we can also define validation rules for metadata field values such as bigger, smaller than a required number, or the fact that a subfield might be optional or mandatory, regex expression defining acceptable values. This is already implemented and present on the HEAP testbed.

As work in progress, HEAP is working on:

- Allowing composition and reusability of the schemas involved in the schematized tags mechanism
- In addition to the search mechanism we provide, we will provide secured access to the metadata index for more complex user defined queries through Kibana.

Seeing that many existing data analysis pipelines from the project involve custom native libraries, which are not fully supported in Hopsworks, we are currently working on providing support for running custom docker images, where the researchers can set up their own custom environments and run their applications.

### Progress and future development and measurements

HEAP platform will deliver exposome data and an agnostic and deterministic assessment of the exposome and its importance. Bioinformatics, machine learning, and advanced statistics will be implemented into the PaaS and tested on the use case cohorts. At the moment, one single instance of HEAP PaaS is deployed on the CSC IaaS as a proof of concept of integration of the PaaS and the IaaS. Metagenomics pipelines and machine learning approaches are being implemented to analyze HEAP’s cohorts.

### Main challenges

#### Fragmentation of research

A major challenge is to overcome fragmentation of research between countries, research institutions, and universities, as well as to overcome the formal obstacles that may be raised by different rules for managing and sharing data. In the case of the former challenge, the integration of innovative approaches within learning healthcare environments constitutes a cornerstone of the so-called next generation medical systems.^38^ However, for this added value to be realized, the transdisciplinary nature of HEAP needs to be implemented considering the various limitations. Thus, the generic platform allows for the creation of different collaborative networks, with standardization implemented at the scientific production-level for consistent and actionable knowledge.

Regarding the challenge for managing and sharing data, HEAP will make a substantial effort into investigating the ethical and legal basis for collaborative international exposome research, by engaging a partner with well-known lawyers with expertise in this area, for example, on the implications of the General Data Protection Regulation on international research.^39^ In collaboration with other ethical and legal contacts in the European Human Exposome Network, HEAP can also serve as a learning exercise in developing generic tools and governance frameworks for exposome research and FAIR data stewardship platforms.^40^

### Communication of research

The innovation in healthcare must be a human-centric process where sophisticated distributed scientific platforms and services are utilized. However, for this to be achieved, it would need to be explained and understood by the users of the platforms (e.g., scientists), as well as the end-users of the generated information (e.g., policymakers, patients). HEAP has created a set of wide-reach communication channels:

- https://heap-exposome.eu/
- https://www.humanexposome.eu/
- https://twitter.com/heap_exposome

Addressing the training and educational component of the research communication challenge, HEAP uses existing educational platforms at IARC, so that HEAP-based educational outputs become immediately globally accessible.^41^ In anticipation of HEAP being utilized by a wide range of potential stakeholders, several “end-user personas” have been created in consultation with the consortium members, so that the end-user characteristics are considered during the platform and services design. This methodology has been well-established and leads to a greater eventual adoption of technological innovation.^42,43^

## Conclusions

The guiding principles of HEAP are that (i) research is international, and global state-of-the-science expertise should be used regardless of the origin of the data, and (ii) exposome studies need to be both large-scale, population-based, continuously ongoing and to be systematically assessed for development of health or disease. Using a limited number of cohorts that are systematically exploited by an international team of researchers with complementary expertise we hope to be able to provide a pilot example of efficient and informative exposome research.

A reproducible and scalable research resource that integrates informatics infrastructures and software to manage, analyze, and produce knowledge in a systematic, flexible, and standardized way, will be a relevant contribution to the exposome research collaboration and the sustainability of the European exposome network.

## Collaboration

HEAP will enable international collaboration to make possible that scientists from different countries can use the same computational platform, following the same ethicolegal requirements, and managing and sharing data in a standardized and efficient way. All the data and knowledge produced by the pilot projects involves close collaboration of all partners from different countries. Within the European Human Exposome Network, HEAP is participating in the working groups for Information and Communication, including managing the joint web site www.humanexposome.eu; on data standardization through the Metadata working group and will be summoning the ethicolegal working group.

## Acknowledgments

The helpful assistance from the members of the European Human Exposome Network is gratefully acknowledged.
